# Enhancement of distribution system performance with reconfiguration, distributed generation and capacitor bank deployment

**DOI:** 10.1016/j.heliyon.2024.e26343

**Published:** 2024-02-17

**Authors:** T. Jayabarathi, T. Raghunathan, N. Mithulananthan, S.H.C. Cherukuri, G. Loknath Sai

**Affiliations:** a School of Electrical Engineering, Vellore Institute of Technology, Vellore, India; b School of Information Technology & Electrical Engineering, The University of Queensland, Brisbane, Australia; c Marelli India Pvt. Ltd., Bangalore, India. Formerly with Power Systems and Smart Grid Laboratory, Indian Institute of Technology, Gandhinagar, India

**Keywords:** Distributed generation, Optimal reconfiguration, Capacitor placement, Metaheuristic optimization

## Abstract

This paper presents a comparative study of optimal reconfiguration, distributed generation, and shunt capacitor bank deployment for power loss minimization and voltage profile improvement in distribution systems. A metaheuristic approach based on the grey wolf optimizer (GWO) algorithm has been proposed for solving this high-dimensional, nonlinear, constrained, combinatorial optimization problem. Two standard IEEE 33- and 69-bus radial distribution systems (RDSs), and a practical 83-bus RDS of Taiwan Power Company have been considered for this study. The solutions obtained are compared with one another and those in the recent literature which includes classical and non-classical, metaheuristic-based methods. Going further, the little-studied problem of simultaneous reconfiguration, distributed generation, and capacitor bank deployment has been solved. The results suggest that the GWO has excellent potential for solving complicated optimization problems in distribution systems and elsewhere.

## Highlights

• The generalized problem dealt with – the optimal reconfiguration/distributed generation/capacitor placement – is highly nonlinear, and discrete.
• It has a number of equality and inequality constraints.
• The grey wolf optimizer, a relatively new metaheuristic optimization algorithm, has been used.
• The algorithm has been demonstrated to be applicable to a high dimensional problem of a practical 83-bus distribution system.
• Extensive comparisons against results from many sources have been provided, to serve as a one-point source of many results and comparisons.

## 1 Introduction

A distribution system is an electrical connecting network between the end consumers and the transmission system. The operation of a distribution system can be formulated as a general optimization problem that can be used to reflect more specific subproblems like optimal network reconfiguration, distributed generation (DG), and capacitor bank (CB) placement or deployment at optimal locations or sites. This is a complicated, nonlinear, non-differentiable, combinatorial optimization problem with many constraints, that are often difficult to solve directly by classical methods [[1], [2], [3]].

The subproblem of optimal distribution system reconfiguration (DSR) can be explained as follows: the distribution system contains two types of switches: the normally closed sectionalizing switches, and the normally open tie-switches. Reconfiguration involves altering the topology of the radial distribution system (RDS) by altering the open or closed status of these switches and maintaining the radiality of the system. DSR can also be combined with the DG deployment subproblem or CB deployment subproblem. The existing literature on the DSR with DG and/or CB deployment falls into four categories: (1) only reconfiguration, (2) reconfiguration with DG deployment, (3) reconfiguration with CB deployment, and (4) reconfiguration with DG and CB deployment. However, studies comparing the performance of these four categories against one another are lacking. Hence, the motivation for this paper is to consider all these categories in one paper and study their relative impact on minimizing power loss and improving the voltage profile using two standard IEEE test systems and a practical system of a power company. This paper further extends the gains made by the inclusion of either DG or CB deployment with reconfiguration by including both DG and CB deployment with reconfiguration. Though it is intuitively evident that such a simultaneous combination of reconfiguration, DG, and CB deployment would provide the best performance in terms of reducing the loss and improving the voltage profile, such a study is lacking in the current literature. This paper provides extensive comparisons against results from many sources, thereby aiming to serve as a one-point source of many results and comparisons.

### 1.1 Only reconfiguration

Methods for solving optimal reconfiguration problems can be broadly classified under the two categories as: classical methods and non-classical, metaheuristic algorithms. Reconfiguration using the classical methods include branch and bound technique [4], 0–1 binary integer programming [5], modified simplex method [6], mixed integer programming [7], and mixed integer convex programming [8]. Recent applications of metaheuristic algorithms for reconfiguration include theta-modified bat algorithm (MBA) [9], bacterial foraging optimization (BFO) algorithm [10], ant colony search [11], genetic algorithm (GA) [12], modified particle swarm optimization [13], mixed integer hybrid differential evolution (MIHDE) [14], plant growth simulation algorithm (PGSA) [15], cuckoo search algorithm (CSA) [16], and improved cuckoo search algorithm (ICSA) [17].

### 1.2 Reconfiguration with DG’s

DGs deployed at optimal locations in an RDS have the benefits of loss reduction, voltage support, improved power quality and reliability, relieving line overloads, and so on [18]. Simultaneous reconfiguration and DG deployment lead to a higher loss reduction than just reconfiguration alone. A detailed literature review of the reconfiguration of RDSs together with the DG deployment subproblem has been presented in Ref. [19]. Several papers dealing with this subproblem have appeared recently. A mixed-integer LP model can be found in Ref. [20]. An optimal reconfiguration of primary feeders of distribution systems employing the convex relaxation method can be found in Ref. [21]. The metaheuristic algorithms used include harmony search (HSA) [22], teaching-learning based optimization [23], adaptive cuckoo search algorithm (ACSA) [24], fireworks algorithm (FWA) [25], UVDA [26], genetic algorithm-improved switch exchange method (GA-ISEM) [27], heuristic method (HM) [28], genetic algorithm (GA) [29], improved neural network algorithm (INNA) [30], chaotic search group algorithm (CSGA) [31], three-dimensional group search optimization (3D-GSO) [32], improved sine-cosine algorithm (ISCA) [33], coyote algorithm (COA) [34], Stochastic Fractal Search (SFS) [35], Equilibrium Optimization Algorithm, Improved Equilibrium Optimization Algorithm (IEOA) [36], and Intelligent Water Drop Algorithm (IWDA) [37].

### 1.3 Reconfiguration with CB’s

Deploying CBs for loss reduction is similar to deploying DGs to minimize losses, with the difference being that DGs supply both the real and reactive power, whereas CBs supply the reactive power alone. The problem of reconfiguration and CB placement has been solved by the classical technique of MINLP [38]. The metaheuristic techniques used are binary particle swarm optimization (BPSO) [39], HSA [40], ant colony algorithm (ACA) [41], the discrete GA, simulated annealing [42], an oppositional krill herd algorithm [43], fuzzy harmony search algorithm (FHSA) [44].

### 1.4 Reconfiguration with DG’s and CB’s

A combination of both DG and CB deployment using various metaheuristic approaches include the water cycle algorithm (WCA) [45], the hybrid tabu search-genetic algorithm [46], intersect mutation differential evolution (IMDE) [47], multi-objective evolutionary algorithm based on decomposition [48], heuristic technique [49], ICA-GA [50], Modified Group Experience of Teaching Learning Based Optimization Approach (MG-TLBO) [51], Manta Ray Foraging Optimization Algorithm (MRFOA) [52], Improved Tunicate Swarm Algorithm (ITSA) [53], and Enhanced Genetic Algorithm (EGA) [54].

The problem of simultaneous reconfiguration with DGs and CBs has been solved with metaheuristic algorithms like improved PSO [55], BFO [56], GA [57,58], NSGA-II [59], and by a classical approach [60].

As the review above indicates, there is a common thread running through these four types of optimization subproblems in RDSs. In this paper, the grey wolf optimizer (GWO) [61], one of the latest metaheuristic algorithms, is used for optimal, simultaneous reconfiguration with DG and CB deployment. So far, the GWO has been implemented for economic dispatch (ED) [62], optimal DG deployment problems [63], and reconfiguration of distribution systems [64].

The main contributions of this paper are: (i) Optimization of reconfiguration and DG deployment, (ii) Optimization of reconfiguration and CB deployment, (iii) Simultaneous optimization of reconfiguration with DG and CB deployment, (iv)Comparative study of each of these optimization subproblems on loss minimization and voltage profile improvement, and (v) Demonstration of the significant loss reduction obtained by simultaneous reconfiguration, DG and CB deployment, (vi) Demonstration of the effectiveness of the GWO as an optimizer for solving the DSR problems, and finally, (vii) extensive comparisons against results from many sources, thereby aiming to serve as a one-point source of many results and comparisons.

This paper is organized as follows: Section 2 defines the problem formulation, and Section 3 overviews the grey wolf optimizer algorithm. Section 4 explains the solution methodology, followed by the application to test cases in Section 5, and the Conclusion in Section 6.

## 2 Problem formulation

The objective or cost function of the optimization problem, and the constraints are outlined next.

### 2.1 Objective function

The aim is to minimize the total power loss by simultaneous optimization of network reconfiguration, optimal placement and sizing of DGs and CBs, without violating the operational constraints required to maintain the system integrity.

**Fig. 1:**
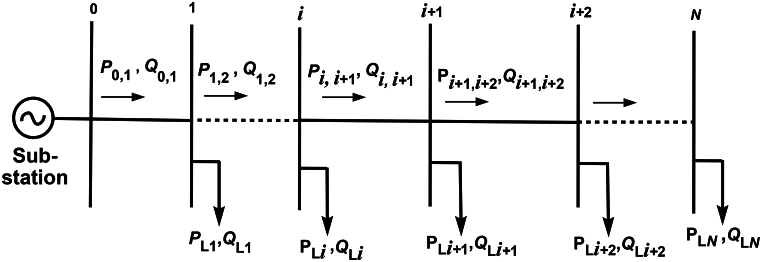
Single-line diagram of an RDS.

Fig. 1 shows the single-line diagram of the main feeder of a radial distribution system (RDS) with *N* number of buses. The real power or $I^{2}R$ loss in the line section between buses *i* and *i*+1 is given by [11,14,15,63]

(1) $$P_{i,i+1}^{\text{Loss}}=\frac{P_{i,i+1}^{2}+Q_{i,i+1}^{2}}{{\left|V_{i}\right|}^{2}}R_{i,i+1}\text{.}$$

The total power loss of the feeder, which is the sum of losses in all the line sections of the feeder, is given by

(2) $$P^{T\text{Loss}}=\sum_{i=0}^{N-1}\frac{P_{i,i+1}^{2}+Q_{i,i+1}^{2}}{{\left|V_{i}\right|}^{2}}R_{i,i+1}$$

where $P_{i,i+1},{\;Q}_{i,i+1}$ are the real and reactive power flow between buses *i* and *i*+1, in kW and kVAr, $R_{i,i+1}$ is the resistance of the line between the buses *i* and *i*+1, and *V*_*i*_ is the voltage at bus *i*.

Similarly, the reactive power loss in the line section between buses *i* and *i*+1 is given by

(3) $$Q_{i,i+1}^{\text{Loss}}=\frac{P_{i,i+1}^{2}+Q_{i,i+1}^{2}}{{\left|V_{i}\right|}^{2}}X_{i,i+1}$$

where $X_{i,i+1}$ is the reactance of the line between buses *i* and *i*+1.

### 2.2.Constraints

1) The bus voltages must be within the admissible bounds:

(4) $$V_{i,\min}\leq V_{i}\leq V_{i,\max}$$

2) The current flow through any distribution line too should be within the upper bound:

(5) $$\left|I_{i,i+1}\right|\leq \left|I_{i,i+1,\max}\right|$$

3) The DG sizes must be within the bounds given by:

(6) $$P_{\text{DG},i,\min}\leq P_{\text{DG},i}\leq P_{\text{DG},i,\max}$$

4) At every bus in the system, the following nonlinear equality constraints must be satisfied (see Fig. 1) [11,14,15,63]:

(7) $$P_{i+1,i+2}=P_{i,i+1}-P_{i,i+1}^{\text{Loss}}-P_{L,i+1}+P_{\text{DG},i+1}$$

(8) $$Q_{i+1,i+2}=Q_{i,i+1}-Q_{i,i+1}^{\;\text{Loss}}-Q_{L,i+1}+Q_{\text{CB},i+1}$$

(9) $${\left|V_{i+1}\right|}^{2}={\left|V_{i}\right|}^{2}-2\left(R_{i,i+1}P_{i,i+1}+X_{i,i+1}Q_{i,i+1}\right)+\left(R_{i,i+1}^{2}+X_{i,i+1}^{2}\right)\left(\frac{P_{i,i+1}^{2}+Q_{i,i+1}^{2}}{{\left|V_{i}\right|}^{2}}\right)$$

where $P_{L,i+1}$ and $Q_{L,i+1}$ are the real and reactive power loads at bus *i*+1. $P_{\text{DG},i+1}$ and $Q_{\text{CB},i+1}$ are the real and reactive power injections by DG and capacitor bank, respectively, at bus *i*+1.

5) Only discrete sizes of capacitors are available in the market. If $Q_{o}^{c}$ is the smallest size, then the set of sizes of capacitors is given by $Q_{o}^{\;c},2Q_{o}^{\;c},...,LQ_{o}^{\;c}\text{,}$ where $LQ_{o}^{\;c}$ is the largest capacitor size.
6) Radiality constraint: For reconfiguration problems, the system radiality has to be maintained. This constraint is expressed as

(10) $$\begin{array}{c} \;\det\;\left(A\right)=0\;\left(\text{non}-\text{radial}\;\text{system}\right)\\ \;\det\;\left(A\right)=1\;\text{or}\;-1\;\left(\text{radial}\;\text{system}\right) \end{array}\}$$

where *A* is the bus incidence matrix.

## 3 The grey wolf optimizer

Proposed by Ref. [61], the grey wolf optimizer (GWO) is a recent metaheuristic optimization method. Like all other metaheuristic methods, GWO starts with an initial swarm of trial solutions, and improves the solutions by mimicking the hunting behavior of grey wolves, modeled by the following equations [62]:

(11) $$\vec{D}=\left|\vec{X}\left(t)-(2{\vec{r}}_{1}\right)⊗{\vec{X}}^{p}\left(t\right)\right|$$

(12) $$\vec{A}=\left(2{\vec{r}}_{2}-1\right)\;a$$

(13) $$\vec{X}\left(t+1\right)={\vec{X}}^{p}\left(t\right)-\vec{D}⊗\vec{A}$$

(14) $${\vec{D}}_{\alpha }=\left|\vec{X}\left(t\right)-\left(2{\vec{r}}_{1}\right)⊗{\vec{X}}^{\alpha }\left(t\right)\right|,{\vec{D}}_{\beta }=\left|\vec{X}\left(t\right)-\left(2{\vec{r}}_{1}\right)⊗{\vec{X}}^{\beta }\left(t\right)\right|,{\vec{D}}_{\delta }=\left|\vec{X}\left(t)-(2{\vec{r}}_{1}\right)⊗{\vec{X}}^{\delta }\left(t\right)\right|$$

(15) $${\vec{X}}_{1}={\vec{X}}^{\alpha }‐{\vec{A}}_{1}⊗{\vec{D}}_{\alpha },{\vec{X}}_{2}={\vec{X}}^{\beta }‐{\vec{A}}_{2}⊗{\vec{D}}_{\beta },{\vec{X}}_{3}={\vec{X}}^{\delta }‐{\vec{A}}_{3}⊗{\vec{D}}_{\delta }$$

(16) $$\vec{X}\left(t+1\right)=\frac{{\vec{X}}_{1}+{\vec{X}}_{2}+{\vec{X}}_{3}}{3}$$

where ${\vec{D}}^{\prime }s$ are the distances, ⊗ denotes component-wise multiplication, ${\vec{r}}_{1},{\vec{r}}_{2}$ are random number vectors extracted from a uniform distribution, ${\vec{A}}^{\prime }s$ are the vector values which guide the motion of the wolves, $t,\vec{X},{\vec{X}}^{p}$ are the iteration number, position of the wolf, and position of the prey, respectively, ${\vec{X}}^{\alpha },{\vec{X}}^{\beta }\;\text{and}\;{\vec{X}}^{\delta }$ are the respective locations of the delta, beta and alpha wolves - best solutions - in the solution space. *a* is decreased progressively from 2 to 0 as the iteration number increases.

## 4 Computational procedure for employing GWO to optimal reconfiguration with DG and CB deployment

Simultaneous reconfiguration with DGs and CBs at the right locations results in loss reduction and improvement in voltage profile. The independent variables in this problem are of three types, namely the locations, capacity of DGs and CBs and open switches. These variables form a grey wolf (vector). The system losses and bus voltages are determined by load flow [65] executed for every wolf. The fitness of the wolf is the loss output of the load flow, given by (2). The first three grey wolves that have the best fitnesses (the minimum or least losses) are selected as the alpha, beta, and delta wolves respectively, and correspond to the optimal values of the locations, capacities of the DGs and CBs, and the open switches.

The stepwise procedure to solve the problem using the GWO approach is as follows.

Step 0 **generation of initial population:** Choose the number of locations for DGs and CBs to be erected, their capacities and open switches. Also choose maximum number of iterations, iter_max_, and population size *N*_*P*_*.*

Generate the initial population of feasible solution vectors (or wolves), that satisfies all the constraints in Section II.B. For 3 locations of DGs and CBs each, and 5 open switches, the matrix of the initial population is given by

(17) $$P=\left[\begin{array}{c} P^{1}\\ P^{2}\\ \vdots \\ P^{N_{P}} \end{array}\right]=\left[\begin{array}{ccccccccccccccccc} l_{S1}^{1} & l_{S2}^{1} & l_{S3}^{1} & S_{1}^{1} & S_{2}^{1} & S_{3}^{1} & l_{CB1}^{1} & l_{CB2}^{2} & l_{CB3}^{3} & Q_{CB1}^{1} & Q_{CB2}^{2} & Q_{CB3}^{3} & SW_{1}^{1} & SW_{2}^{1} & SW_{3}^{1} & SW_{4}^{1} & SW_{5}^{1}\\ l_{S1}^{2} & l_{S2}^{2} & l_{S3}^{3} & S_{1}^{2} & S_{2}^{2} & S_{3}^{2} & l_{CB1}^{2} & l_{CB2}^{2} & l_{CB3}^{3} & Q_{CB1}^{1} & Q_{CB2}^{2} & Q_{CB3}^{3} & SW_{1}^{1} & SW_{2}^{1} & SW_{3}^{1} & SW_{4}^{1} & SW_{5}^{1}\\ \vdots & \vdots & \vdots & \vdots & \vdots & \vdots & \vdots & \vdots & \vdots & \vdots & \vdots & \vdots & \vdots & \vdots & \vdots & \vdots & \vdots \\ l_{S1}^{N_{P}} & l_{S2}^{N_{P}} & l_{S3}^{N_{P}} & S_{1}^{N_{P}} & S_{2}^{N_{P}} & S_{3}^{N_{P}} & l_{CB1}^{N_{P}} & l_{CB2}^{N_{P}} & l_{CB3}^{N_{P}} & Q_{CB1}^{N_{P}} & Q_{CB2}^{N_{P}} & Q_{CB3}^{N_{P}} & SW_{1}^{N_{P}} & SW_{2}^{N_{P}} & SW_{3}^{N_{P}} & SW_{4}^{N_{P}} & SW_{5}^{N_{P}} \end{array}\right]$$

where *l* is the bus number or location, *S*, *Q*, are the capacity of the DG and CB respectively, *SW* are the open tie-switches in the loops *L*_1_ to *L*_5_.

Step 1 **calculation of fitness:** Find the power loss in the RDS for each wolf, by running the load flow. Determine fitness using the cost function given by (2). Spot the alpha, beta and delta wolves.
Step 2 **creation of new population:** Use the encircling operator (14) to calculate

(18) $${\vec{D}}_{\alpha }=\left|{\vec{P}}^{i}-\left(2{\vec{r}}_{3}\right)⊗{\vec{P}}^{\alpha }\right|,{\vec{D}}_{\beta }=\left|{\vec{P}}^{i}-\left(2{\vec{r}}_{4}\right)⊗{\vec{P}}^{\beta }\right|,{\;\vec{D}}_{\delta }=\left|{\vec{P}}^{i}-\left(2{\vec{r}}_{5}\right)⊗{\vec{P}}^{\delta }\right|$$

where the *r*'s are random number vectors ∈ [0, 1].

Calculate ${\vec{P}}_{1},{\vec{P}}_{2}\;\text{and}\;{\vec{P}}_{3}$ using (15). These can be computed as

(19) $${\vec{P}}_{1}={\vec{P}}^{\alpha }-{\vec{A}}_{1}⊗{\vec{D}}_{\alpha },{\;\vec{P}}_{2}={\vec{P}}^{\beta }-{\vec{A}}_{1}⊗{\vec{D}}_{\beta },{\vec{P}}_{3}={\vec{P}}^{\delta }-{\vec{A}}_{1}⊗{\vec{D}}_{\delta }\text{.}$$

Calculate the swarm of the next generation using the hunting operator (16):

(20) $$\vec{P}\left(t+1\right)=\frac{{\vec{P}}_{1}+{\vec{P}}_{2}+{\vec{P}}_{3}}{3}$$

Step 3 **checking of constraints:** Check if the limits of the constraints are violated. If a violation occurs, fix at the limit violated, and add a penalty for the violation.
Step 4 Repeat steps 1 to 3 until the criterion for stopping is satisfied, or for the specified number of iterations.

## 5 Test cases, results, and discussion

The GWO approach reported in this paper is applied and tested on three test cases comprising IEEE 33- and IEEE 69-bus, and a practical, Taiwan Power Company (TPC) 83-bus RDSs. The bounds of DG sizes chosen for deployment are 0 and 2 MW for the first two test cases, and 0 and 3.5 MW for the third test case. The DGs and CBs connected at a bus are modeled as negative real and reactive power loads in the corresponding load bus.

The DGs installed inject only real power, and the CBs inject reactive power. The maximum and minimum bus voltage limits for all the test cases are *V*_max_ = 1.05 p. u. and *V*_min_ = 0.9 p. u. [22,28]. For the load flow solution, the direct approach proposed by Teng [65] has been used. For determining the connectivity matrix for the reconfiguration of the radial system, biograph theory has been used [66].

The methodology for reconfiguration begins with all the switches closed, in the meshed distribution network. The switches are opened one by one, to eliminate loops. The criterion for opening a switch is the minimization of total power loss. That switch which produces minimum power loss should be opened.

The GWO-based method and the load flow solution are executed on a 3.0 GHz, 64-bit personal computer with an i7 processor and 8 GB RAM, using MATLAB® software. The number of wolves is fixed at 40, and the total number of iterations is fixed as 200. With these parameters, three test cases are considered in this paper.

For all the test cases, the following scenarios are studied, and comparisons done against the results available in the recent literature.

Scenario 1: Optimal reconfiguration and DG deployment (location and sizing):
  Case (i) Only reconfiguration.
  Case (ii) Only DG deployment.
  Case (iii) DG deployment after reconfiguration.
  Case (iv) Simultaneous reconfiguration and DG deployment.
Scenario 2: Optimal reconfiguration and CBs deployment (location and sizing):
  Case (i) Only reconfiguration.
  Case (ii) Only CB deployment.
  Case (iii) CB deployment after reconfiguration.
  Case (iv) Simultaneous reconfiguration and CB deployment.
Scenario 3: Optimal reconfiguration with DG and CB deployment.
  Case (i) Only reconfiguration.
  Case (ii) DG and CB deployment.
  Case (iii) Simultaneous reconfiguration with DG and CB deployment.

### 5.1 IEEE 33-BUS RDS

This system has 5 tie switches, 32 sectionalizing switches, 37 lines, and 33 buses. The 5 tie switches are the line segments 33, 34, 35, 36 and 37. These form 5 loops L_1_ to L_5_. The total reactive and real power demands are 2300 kVAr and 3715 kW respectively [22,24,28,29]. With the base values as *V*_base_ = 12.66 kV, *S*_base_ = 10 MVA, the per unit system of calculations is used. The low voltages at the end buses of this RDS - caused by inductive loads - can be mitigated by connecting to these buses CBs and DGs to supply locally part of the reactive and real power demand. Consequently, the current flows and losses are reduced.

The base case or uncompensated power loss is 202.66 kW for this system. In the tabulations, results obtained by other methods are compared with those papers whose base case loss is 202.66 kW.

Table 1 shows the status of the switches opened for reconfiguration, DG sizes and their locations (bus no. s), minimum bus voltages and power losses for Scenario 1 of the IEEE 33-bus RDS, obtained using the GWO approach proposed in this paper. The results reported in the literature by other methods are also shown for comparison, for each of the Cases under this Scenario.

**Table 1:** Scenario 1: Reconfiguration and DG deployment for IEEE 33-bus RDS.

Method	Switches opened	DG size, kW (bus number)	Min. voltage (p.u.)	Power loss, kW
Base case	33, 34, 35, 36, 37	–	0.9131	202.66
Case (i): only reconfiguration				
**GWO**	**7, 14, 9, 32, 37**	**----**	**0.9378**	**139.55**
MBA [9]	7, 9, 14, 32, 37	–	–	139.53
ICSA [17]	7, 14, 9, 32, 37	–	0.9378	139.55
HSA [22]	7, 14, 9, 32, 37	–	0.9378	139.55
ACSA [24]	7, 14, 9, 32, 28	–	0.9413	139.55
FWA [25]	7, 14, 9, 32, 28	–	0.9413	139.98
UVDA [26]	7, 9, 14, 32, 37	–	0.9378	139.55
HM [28]	7, 9, 14, 32, 37	–	0.9378	139.55
INNA [30]	7, 9, 14, 32, 37	–	0.9378	139.55
3D-GSO [32]	7, 9, 14, 28, 32	–	0.9439	139.26
ISCA [33]	7, 14, 9, 32, 37	–	0.9378	139.55
SFS [35]	7, 14, 9, 32, 37	–	0.9378	139.55
IWDA [37]	7, 9, 14, 32, 37	–	0.9465	139.48
ACA [41]	7, 9, 14, 32, 37	–	0.9375	139.68
Case (ii): only DG deployment				
**GWO**	**33, 34, 35, 36, 37**	**753.7 (13), 1101 (23), 1072 (29)**	**0.9723**	**71.45**
HSA [22]	33, 34, 35, 36, 37	107.0 (18), 572.4 (17), 1046.2 (33)	0.9670	96.76
ACSA [24]	33, 34, 35, 36,37	779.8 (14), 1125.1 (24), 1349.5 (30)	0.9778	74.26
FWA [25]	33, 34, 35, 36, 37	589.7 (14), 189.5 (18), 1014.6 (32)	0.9680	88.68
UVDA [26]	33, 34, 35, 36, 37	875 (11), 925 (29), 931 (24)	0.9620	74.21
HM [28]	33, 34, 35, 36, 37	740.6 (14), 1009.4 (24), 1054.2 (30)	0.9676	71.50
INNA [30]	33, 34, 35, 36, 37	705.0 (14), 570.2 (25), 953.8 (30)	0.9622	75.42
3D-GSO [32]	33, 34, 35, 36, 37	766 (11), 285.2 (18), 903.3 (32)	0.9928	79.87
ISCA [33]	33, 34, 35, 36, 37	743.0 (14), 743.0 (24), 743.0 (31)	0.9612	77.13
SFS [35]	33, 34, 35, 36, 37	754.0 (14), 1099.4 (24), 1071.4 (30)	0.9687	71.47
IWDA [37]	33, 34, 35, 36, 37	105.9 (8), 552.6 (29), 1045.8 (24)	0.9619	70.42
WCA [45]	33, 34, 35, 36, 37	854.6 (14), 1101.7 (24), 1181.0 (29)	0.9730	71.05
Case (iii): DG deployment after reconfiguration				
**GWO**	**7, 14, 9, 32, 37**	**943.8 (8), 1092.6 (24), 950.5 (30)**	**0.9740**	**58.88**
HSA [22]	7, 14, 9, 32, 37	268.6 (32), 161.1 (31), 661.2 (30)	0.9479	97.13
ACSA [24]	7, 14, 9, 32, 28	1753.6 (29), 539.7 (12), 504.5 (16)	0.9802	58.79
FWA [25]	7, 14, 9, 32, 28	599.6 (32), 314.1 (33), 159.1 (18)	0.9612	83.91
UVDA [26]	7, 9, 14, 32, 37	1125 (30), 592 (15), 526 (12)	0.9758	66.60
HM [28]	7, 9, 14, 32, 37	920.8 (8), 1054.5 (24), 924.2 (30)	0.9733	58.90
3D-GSO [32]	7, 14, 9, 32, 28	658.9 (31), 724.7 (12), 556.8 (33)	0.9923	71.21
SFS [35]	7, 9, 14, 32, 37	1068.2 (24), 950.3 (30), 931.7 (8)	0.9741	58.88
IWDA [37]	37, 32, 14, 9, 7	257.8 (30), 162.3 (15), 742.1 (12)	0.9757	58.47
Case (iv): simultaneous reconfiguration and DG deployment				
**GWO**	**33, 34, 11, 30, 28**	**986.2 (7), 855.9 (18), 1166.6 (25)**	**0.9673**	**50.90**
HSA [22]	7, 14, 10, 32, 28	525.8 (32), 558.6 (31), 584.0 (33)	0.9700	73.05
ACSA [24]	7, 10, 13, 32, 27	426.3 (32), 1202.4 (29), 712.71 (18)	0.9786	63.69
FWA [25]	7, 14, 11, 32, 28	536.7 (32), 615.8 (29), 531.5 (18)	0.9713	67.11
UVDA [26]	7, 10, 13, 27, 32	1554 (29), 649 (15), 486 (21)	0.9760	57.28
HM [28]	11, 28, 30, 33, 34	899.7 (7), 865.1 (18), 1295.6 (25)	0.9680	51.30
INNA [30]	7, 9, 14, 27, 30	482.2 (12), 1015.3 (25), 731.5 (33)	0.9674	54.69
CSGA [31]	7, 9, 14, 28, 30	469.7 (12), 2021.3 (25), 7380 (33)	0.9677	54.48
3D-GSO [32]	7, 8, 14, 25, 36	630.0 (12), 600.0 (18), 1190.0 (30)	0.9899	57.97
ISCA [33]	7, 9, 14, 28, 31	648.5 (30), 510.3 (13), 532.5 (16)	0.9611	66.81
SFS [35]	7, 9, 14, 27, 30	775.3 (22), 735.6 (33), 1285.8 (25)	0.972	53.01
IEOA [36]	7, 9, 14, 27, 31	421 (12), 650 (18), 1158 (29)	0.9748	55.60
IWDA [37]	32, 28, 14, 11, 7	1583.1 (29), 672.3 (15), 501.7 (21)	0.9764	55.24
BFO [56]	28, 20, 17, 10, 14	795 (13), 1069 (24), 1029 (30)	0.9728	72.26

As seen from the table, for Case (i) (only reconfiguration), the GWO yields a power loss of 139.55 kW, which is almost the same as those by other methods. For Case (ii) (only DG deployment), the GWO yields a power loss of 71.45 kW, which is the lowest of all methods, except the WCA [45], and IWDA [37]. For Case (iii) (DG deployment after reconfiguration), the GWO yields a marginally higher figure than that by ACSA [24], and IWDA [37]. For Case (iv) (Simultaneous reconfiguration and DG deployment), the GWO returns a power loss of 50.90 kW, which is the lowest of all methods.

Table 2 shows the comparison of the outcomes obtained for Scenario 2, optimal reconfiguration and CBs deployment (location and sizing). The first two rows of Table 1, showing only the base case, and only reconfiguration (Case (i), in all the Scenarios 1, 2 and 3), have been repeated in Table 2, Table 3, for ready reference. For Case (ii) (only CB deployment), the GWO yields the least power loss of 132.29 kW, except WCA [45], which yields 130.91 kW. For Case (iii), the GWO is better than the only other reported result, BPSO [39]. For Case (iv), the GWO yields a power loss of 92.59 kW, which is the lowest of all.

**Table 2:** Scenario 2: Simultaneous reconfiguration and CB deployment for IEEE 33-bus RDS.

Method	Switches opened	Capacitor size, kVAr (bus number)	Min. voltage (p.u.)	Power loss, kW
Base case	33, 34, 35, 36, 37	–	0.9131	202.66
Case (i): only reconfiguration				
**GWO**	**7, 14, 9, 32, 37**	**----**	**0.9378**	**139.55**
Case (ii): only CB deployment				
**GWO**	**33, 34, 35, 36, 37**	**534 (23), 1100 (29), 363 (12)**	**0.9378**	**132.29**
BPSO [39]	33, 34, 35, 36, 37	3300 kVAr at 7 locations	0.9389	134.20
HSA [40]	33, 34, 35, 36, 37	900 (6), 300 (28), 600 (29), 300 (30)	0.9349	135.16
ACA [41]	33, 34, 35, 36, 37	600 (9), 450 (28), 600 (29)	0.9387	136.14
WCA [45]	33, 34, 35, 36, 37	397.3 (14), 451.1 (24), 1000 (30)	0.9510	130.91
Case (iii): CB deployment after reconfiguration				
**GWO**	**7, 14, 9, 32, 37**	**472 (24), 1100 (30), 579 (21)**	**0.9555**	**93.29**
BPSO [39]	7, 9, 14, 32, 37	1200 (4), 900 (7), 1800 (8), 1200 (9), 300 (16)	0.9612	94.26
Case (iv): simultaneous reconfiguration and CB deployment				
**GWO**	**7, 14, 9, 32, 37**	**533 (24), 957 (30), 445 (8)**	**0.9595**	**92.59**
BPSO [39]	7, 9, 14,32, 37	600 (7), 300 (12), 300 (25), 600 (30), 300 (33)	0.9585	93.06
HSA [40]	33, 14, 8, 32, 28	900 (6), 300 (28), 600 (29), 300 (30), 300 (9)	0.9411	119.72
ACA [41]	7, 9, 14, 32, 37	600 (20), 450 (28), 600 (29)	0.9656	95.79
BFO [56]	7, 11, 34, 37, 36	600 (5), 300 (16), 300 (25)	0.9712	101.08

**Table 3:** Scenario 3: Simultaneous reconfiguration with DG and CB deployment for IEEE 33-bus RDS.

Method	Switches opened	DG size, kW (bus number)	Capacitor size, kVAr (bus number)	Min. voltage (p.u.)	Power loss, kW
Base case	33, 34, 35, 36, 37	–	–	0.9131	202.66
Case (i): only reconfiguration					
**GWO**	**7, 14, 9, 32, 37**	**----**	**-----**	**0.9378**	**139.55**
Case (ii): DG and CB deployment					
**GWO**	**33, 34, 35, 36, 37**	**797 (12), 1114 (29), 824 (24)**	**372 (13), 1038 (29), 545 (23)**	**0.9934**	**12.47**
WCA [45]	33, 34, 35, 36, 37	973 (25), 1040 (29), 563.0 (11)	465 (23), 565 (30), 535 (14)	0.9800	24.68
MRFOA [52] ITSA [53]	33, 34, 35, 36, 37 33, 34, 35, 36, 37	803 (13), 1073 (24), 1040 (30) 788 (13), 742 (25), 1085 (30)	300 (14), 600 (24), 900 (30) 834 (30), 603 (7), 269 (15)	–	12.57 14.40
EGA [54]	33, 34, 35, 36, 37	1095 (24), 767.7 (14), 964.2 (30)	389 (25), 335 (14), 1190 (30)	0.9924	12.7
GA [58]	33, 34, 35, 36, 37	250 (16), 250 (22), 500 (30)	1800 kVAr at 5 loc.	0.9720	71.60
Case (iii): simultaneous reconfiguration with DG and CB deployment					
**GWO**	**5, 14, 35, 17, 26**	**1050.1 (9), 1174.7 (25), 717.8 (33)**	**450 (9), 600 (29), 600 (30)**	**0.9875**	**12.14**
BFO [56]	9, 14, 27, 33, 36	758 (14), 1045 (24), 987 (30)	150 (8), 150 (18), 300 (30)	0.9757	45.65
GA [57]	7, 9, 15, 27, 34	300 (15), 300 (18), 300 (29), 600 (30), 300 (31)	250 (16), 250 (22), 500 (30)	0.9810	50.149
GA [58]	7, 9, 13, 15, 27	250 (16), 250 (22), 500 (30)	1800 kVAr at 5 locations	0.9804	48.00

Table 3 presents the results for Scenario 3 (optimal reconfiguration with DG and CB deployment). For Case (ii) (DG and CB deployment), at 12.47 kW, which is the least of all. For Case (iii) (Simultaneous reconfiguration with DG and CB deployment), the GWO produces results far superior to those in the literature.

### 5.2 IEEE 69-BUS RDS

This RDS comprises 5 tie switches, 68 sectionalizing switches, 73 branches, and 69 buses. The 5 tie switches are the line segments 69, 70, 71, 72 and 73. These form 5 loops L_1_ to L_5_. The base values in per unit (p.u.) are *V*_base_ = 12.66 kV, and *S*_base_ = 10 MVA. The gross reactive and real power demands for this system are 2694.1 kVAr and 3801.89 kW respectively [22,24,28,29]. Buses 12, 11, 9, 7 and 4 have two branches, bus 3 has three branches, and the rest of the buses have just one branch connection to their next bus. The base case power loss for this RDS is 225 kW.

Table 4 displays the status of the switches opened for reconfiguration, DG sizes and their locations (bus no. s), minimum bus voltages and power losses for Scenario 1 of the IEEE 69-bus RDS, obtained using the GWO approach proposed in this paper. Also shown in the table is the comparison with the outcomes obtained by the other approaches in the literature, for each of the Cases under this Scenario. As can be observed from the table, the GWO is the overall best performer, as compared to any other method, except SFS [35].

**Table 4:** Scenario 1: Reconfiguration and DG deployment for IEEE 69-bus RDS.

Method	Switches opened	DG size, kW (bus number)	Min. voltage (p.u.)	Power loss, kW
Base case	69, 70, 71, 72, 73	–	0.9092	225.00
Case (i): only reconfiguration				
**GWO**	**69, 14, 71, 61, 58**	**----**	**0.9492**	**98.63**
ICSA [17]	14, 57, 61, 69, 70	–	0.9495	98.59
HSA [22]	69, 18, 13, 56, 61	–	0.9428	99.35
ACSA [24]	69, 70, 14, 57, 61	–	0.9495	98.59
FWA [25]	69, 70, 14, 56, 61	–	0.9495	98.59
UVDA [26]	14, 58, 61, 69, 70	–	0.9495	98.58
HM [28]	14, 55, 61, 69, 70	–	0.9428	99.62
INNA [30]	14, 57, 61, 69, 70	–	0.9495	98.60
3D-GSO [32]	14, 56, 61, 69, 70	–	0.9498	98.59
ISCA [33]	14, 55, 61, 69, 70	–	0.9495	98.60
COA [34]	69, 70, 14, 57, 61	–	0.9495	98.59
SFS [35]	14, 55, 61, 69, 70	–	0.9495	98.62
IWDA [37]	69, 61, 56, 18, 13	–	0.9489	98.58
Case (ii): only DG deployment				
**GWO**	69, 70, 71, 72, 73	**563.8 (15), 607.14 (50), 1800 (61)**	**0.9795**	**70.27**
HSA [22]	69, 70, 71, 72, 73	101.8 (65), 369.0 (64), 1302.4 (63)	0.9677	86.77
ACSA [24]	69, 70, 71, 72, 73	602.2 (11), 380.4 (18), 2000 (61)	0.9890	72.44
FWA [25]	69, 70, 71, 72, 73	408.5 (65, 1198.6 (61), 225.8 (27)	0.9740	77.85
UVDA [26]	69, 70, 71, 72, 73	1410 (61), 604.0 (11), 417.0 (17)	0.9688	72.62
HM [28]	69, 70, 71, 72, 73	519.3 (17), 719.6 (50), 1726.6 (61)	0.9770	70.30
INNA [30]	69, 70, 71, 72, 73	203.6 (12), 388.5 (18), 1687.0 (61)	0.9760	70.66
3D-GSO [32]	69, 70, 71, 72, 73	388.0 (27), 1464.0 (61), 281.0 (64)	0.9792	73.48
ISCA [33]	69, 70, 71, 72, 73	760.4 (12), 760.4 (61), 760.4 (62)	0.9717	74.40
SFS [35]	69, 70, 71, 72, 73	526.8 (11), 380.4 (18), 1719.0 (61)	0.9790	69.44
IWDA [37]	69, 70, 71, 72, 73	105.9 (65), 552.6 (64), 1045.8 (17)	0.9619	76.42
WCA [45]	69, 70, 71, 72, 73	775.0 (61), 1105.0 (62), 438.0 (23)	0.9870	71.50
Case (iii): DG deployment after reconfiguration				
**GWO**	**69, 14, 71, 61, 58**	**1414.36 (61), 470 (65), 374.5 (66)**	**0.9807**	**35.82**
HSA [22]	69, 18, 13, 56, 61	1066.6 (61), 352.9 (60), 425.7 (58)	0.9619	51.30
ACSA [24]	69, 70, 14, 57, 61	1725.4 (61), 466.6 (64), 368.6 (12)	0.9870	37.23
FWA [25]	69, 70, 14, 56, 61	1001.4 (61), 214.5 (62), 142.5 (64)	0.9720	43.88
UVDA [26]	14, 58, 61, 69, 70	1378.0 (61), 620.0 (11), 722.0 (64)	0.9801	37.84
HM [28]	14, 55, 61, 69, 70	536.7 (11), 1412.8 (61), 483.4 (64)	0.9746	35.94
3D-GSO [32]	14, 56, 61, 69, 70	1000 (61), 441 (62), 752.0 (50)	0.9742	40.60
SFS [35]	14, 55, 61, 69, 70	537.6 (11), 1434.0 (61), 490.3 (64)	0.9813	35.17
IWDA [37]	69, 61, 56, 18, 13	1067 (61), 224.6 (11), 138.4 (64)	0.9757	35.57
Case (iv): simultaneous reconfiguration and DG deployment				
**GWO**	**69, 14, 70, 62, 58**	**537.8 (11), 1480 (61), 489.6 (65)**	**0.9810**	**35.39**
HSA [22]	69, 17, 13, 58, 61	1066.6 (61), 352.5 (60), 452.7 (62)	0.9736	40.30
ACSA [24]	69, 70, 12, 58, 61	1749.6 (61), 156.6 (62), 409.0 (65)	0.9873	40.49
FWA [25]	69, 70, 13, 55, 63	1127.2 (61), 275.0 (62), 415.9 (65)	0.9796	39.25
UVDA [26]	14, 58, 63, 69, 70	1472 (61), 538.0 (11), 673.0 (17)	0.9816	37.111
HM [28]	14, 57, 61, 69, 70	539.4 (11), 1420.4 (61), 489.6 (64)	0.9747	35.42
INNA [30]	14, 55, 61, 69, 70	418.1 (12), 1380.5 (61), 482.7 (64)	0.9802	35.37
CSGA [31]	14, 55, 61, 69, 70	406.2 (12), 1400.4 (61), 474.6 (64)	0.9806	35.35
3D-GSO [32]	14, 56, 61, 69, 70	1313.0 (61), 441.0 (62), 752.0 (50)	0.9823	38.18
ISCA [33]	12, 19, 69, 63, 57	1000.9 (61), 410.6 (62), 461.6 (65)	0.9798	39.73
COA [34]	69, 70, 14, 58, 61	490.2 (64), 537.4 (11), 1434.0 (61)	0.9813	35.15
SFS [35]	14, 56, 61, 69, 70	490.3 (64), 537.6 (11), 1434.0 (61)	0.9810	35.16
EOA [36]	10, 20, 58, 71, 73	539 (22), 1452 (61), 289 (64)	0.9721	44.41
IWDA [37]	69, 61, 58, 17, 13	526.3 (61), 692.1 (17), 1512.4 (11)	0.9801	35.74

Table 5 displays the results obtained for Scenario 2 of the IEEE 69-bus RDS, and the results by other methods. For case (ii), the GWO yields a higher power loss of 145.56 kW, which is marginally greater than the WCA [45], which yields 144.53 kW.

**Table 5:** Scenario 2: Optimal reconfiguration and CB deployment for the IEEE 69-bus RDS.

Method	Switches opened	Capacitor size, kVAr (bus number)	Min. voltage (p.u.)	Power loss, kW
Base case	69, 70, 71, 72, 73	–	0.9092	225.00
Case (i): only reconfiguration				
**GWO**	**69, 14, 71, 61, 58**	**----**	**0.9492**	**98.63**
Case (ii): only CB deployment				
**GWO**	**69, 70, 71, 72, 73**	**697 (8), 260 (18), 1200 (60)**	**0.9319**	**145.56**
WCA [45]	69, 70, 71, 72, 73	1288.2 (61), 213.4 (69), 270.0 (18)	0.9500	144.53
Case (iii): CB deployment after reconfiguration				
**GWO**	**69, 14, 71, 61, 58**	**315 (25), 350 (69), 1100 (61)**	**0.9678**	**66.88**
Case (iv): simultaneous reconfiguration and CB deployment				
**GWO**	**69, 12, 17, 61, 55**	**990 (61), 443 (27), 377 (10)**	**0.9660**	**68.92**
NSGA-II [59]	14, 17, 56, 64, 69	500 (12), 500 (49), 500 (60), 500 (61)	0.9574	79.77

For Case (iii), no reported results are available for comparison. For Case (iv), the GWO is better than the only other reported work.

Table 6 presents the results for Scenario 3 of the IEEE 69-bus system. For Case (ii), the total loss for GWO is 6.19 kW, which is the least of all methods. For Case (iii), the GWO yields a power loss of 7.19 kW, which is again the best result.

**Table 6:** Scenario 3: Reconfiguration with DG and CB deployment for IEEE 69-bus RDS.

Method	Switches opened	DG size, kW (bus number)	Capacitor size, kVAr (bus number)	Min. voltage (p.u.)	Power loss, kW
Base case	69, 70, 71, 72, 73	–	–	0.9092	225.00
Case (i): only reconfiguration					
**GWO**	**69, 14, 71, 61, 58**	**----**	**---**	**0.9492**	**98.63**
Case (ii): DG and CB deployment					
**GWO**	**69, 70, 71, 72, 73**	**455.1 (18), 782.6 (6), 1584.7 (60)**	**1100 (60), 263 (18), 286 (68)**	**0.9938**	**6.19**
WCA [45]	69, 70, 71, 72, 73	540.8 (17), 2000 (61), 1159.2 (69)	1187.9 (2), 1237.3 (62), 269.7 (69)	0.9940	33.339
IMDE [47]	69, 70, 71, 72, 73	1738 (62), 479 (24)	109 (63), 1192 (61)	0.9915	13.83
ITSA [53]	69, 70, 71, 72, 73	291 (10), 491 (15), 1500 (61)	288 (9), 292 (23), 1149 (61)	–	6.80
Case (iii): simultaneous reconfiguration with DG and CB deployment					
**GWO**	**9, 70, 17, 26, 55**	**614.7 (37), 1801.3 (61), 597.5 (22)**	**290 (24), 926 (61), 230 (27)**	**0.9937**	**7.19**
BFO [56]	13, 17, 47, 50, 69	350 (11), 615 (18), 1164 (61)	150 (21), 300 (61), 450 (64)	0.9733	28.87
NSGA-II [59]	20, 37, 43, 57, 61	400 (58), 400 (61), 400 (65)	500 (7), 500 (12), 500 (50), 500 (61)	0.9766	29.748

### 5.3 TPC 83-BUS RDS

This RDS, a practical system of the Taiwan Power Company (TPC), comprises 11 feeders, 13 tie switches, and 83 sectionalizing switches [14,15]. The base case power loss is 531.99 kW. The real and reactive power loads are 28.35 MW and 20.7 MVAR respectively. With the base values as *V*_base_ = 11.40 kV, *S*_base_ = 100 MVA, the per unit system of calculations is used.

Table 7 shows the status of the switches opened for reconfiguration, DG sizes and their locations (bus no. s), minimum bus voltages and power losses for Scenario 1 of the TPC 83-bus RDS, obtained using the GWO approach of this paper. The results reported by other methods are also shown for comparison, for each of the Cases under this Scenario.

**Table 7:** Scenario 1: Reconfiguration and DG deployment for TPC 83-bus RDS.

Method	Switches opened	DG size, kW (bus number)	Min. voltage (p.u.)	Power loss, kW
Base case	84, 85, 86, 87, 88, 89, 90, 91, 92, 93, 94, 95, 96	–	0.9285	531.99
Case (i): only reconfiguration				
**GWO**	**55, 7, 86, 72, 13, 89, 90, 83, 92, 93, 34, 40, 62**	**–**	**0.9531**	**470.10**
GA [12]	55, 7, 86, 72, 13, 89, 90, 83, 92, 39, 34, 41, 62	**–**	0.9532	469.88
MIHDE [14]	55, 7, 86, 72, 13, 89, 90, 83, 92, 39, 34, 41, 62	**–**	0.9532	469.88
PGSA [15]	55, 7, 43, 72, 13, 18, 26, 83, 32, 39, 34, 42, 62	**–**	0.9532	469.88
GA-ISEM [27]	7, 13, 34, 39, 42, 55, 62, 72, 83, 86, 89, 90, 92	**–**	0.9532	469.87
HM [28]	7, 13, 34, 39, 42, 63, 72, 83, 84, 86, 89, 90, 92	**–**	0.9518	470.01
SFS [35]	55, 7, 86, 72, 13, 89, 90, 83, 92, 39, 34, 42, 62	**–**	0.9532	469.88
FHSA [44]	7, 13, 39, 42, 55, 62,72, 83, 86, 89, 90, 91, 92	**–**	0.9532	469.88
Case (ii): only DG deployment				
**GWO**	**84, 85, 86, 87, 88, 89, 90, 91, 92, 93, 94, 95, 96**	**3340 (7), 2340 (72), 3500 (80)**	**0.9572**	**359.76**
HM [28]	84, 85, 86, 87, 88, 89, 90, 91, 92, 93, 94, 95, 96	3104.5 (7), 2516.6 (72), 3599.6 (80)	0.9554	359.2
SFS [35]	84, 85, 86, 87, 88, 89, 90, 91, 92, 93, 94, 95, 96	3584.7 (80), 2835.0 (72), 3138.9 (7)	0.9557	359.73
Case (iii): DG deployment after reconfiguration				
**GWO**	**55, 7, 86, 72, 13, 89, 90, 83, 92, 93, 34, 40, 62**	**3500 (20), 3030 (54), 3200 (80)**	**0.9532**	**345.64**
HM [28]	7, 13, 34, 39, 42, 63, 72, 83, 84, 86, 89, 90, 92	1998.6 (8), 3474.9 (20), 3026.7 (54)	0.9532	345.9
SFS [35]	55, 7, 86, 72, 13, 89, 90, 83, 92, 39, 34, 42, 62	3489.5 (20), 3171.5 (80), 3029.6 (54)	0.9532	345.41
Case (iv): simultaneous reconfiguration and DG deployment				
**GWO**	**84, 7, 86, 72, 76, 89, 90, 83, 92, 36, 34, 40, 61**	**3500 (20), 3100 (54), 2250 (72)**	**0.9561**	**349.30**
HM [28]	14, 34, 39, 42, 54, 64, 81, 85, 86, 87, 90, 93, 94	3781.2 (7), 4700.3 (20), 2518.2 (72)	0.9500	326.36
CSGA [31]	7, 33, 39, 42, 61, 70, 84, 86, 88, 89, 90, 91, 92	3251.2 (54), 3634.1 (72), 3585.3 (80)	0.9561	342.30
SFS [35]	84, 85, 86, 72, 88, 14, 90, 81, 92, 39, 34, 42, 62	3138.9 (7), 4754.8 (20), 3231.2 (54)	0.9532	327.83

As seen from the table, for the Case (i) (only reconfiguration), the GWO yields a power loss of 470.10 kW, which is comparable to those by other methods. For Case (ii) (only DG deployment), the GWO yields a power loss of 359.76 kW, which is comparable to that by SFS [35]. For Case (iii) (DG deployment after reconfiguration), the GWO yields a 345.64 kW, which is comparable to that by other methods. For Case (iv) (Simultaneous reconfiguration and DG deployment), the GWO returns a power loss of 349.30 kW, which is a worse result than those by other methods.

Table 8 shows the comparison of the outcomes obtained for Scenario 2, optimal reconfiguration and CBs deployment (location and sizing). The first two rows of Table 7, showing only the base case, and only reconfiguration (Case (i), in all the Scenarios 1, 2 and 3), have been repeated in Table 8, Table 9, for ready reference. For Case (ii) (only CB deployment), the GWO yields a power loss of 413.67 kW, which is a significant improvement over the other method which yields 430.08 kW. For Case (iii), there is no work available for comparison. For Case (iv), the GWO yields a power loss of 368.11 kW, lower than 375.71 kW by the other method.

**Table 8:** Scenario 2: Optimal reconfiguration and CBs deployment for TPC 83-bus RDS.

Method	Switches opened	Capacitor size, kVAR (bus number)	Min. voltage (p.u.)	Power loss, kW
Base case	84, 85, 86, 87, 88, 89, 90, 91, 92, 93, 94, 95, 96	–	0.9285	531.99
Case (i):only reconfiguration				
**GWO**	**55, 7, 86, 72, 13, 89, 90, 83, 92, 93, 34, 40, 62**	**–**	**0.9531**	**470.10**
Case (ii):only CB deployment				
**GWO**	**84, 85, 86, 87, 88, 89, 90, 91, 92, 93, 94, 95, 96**	**1350 (6), 900 (18), 150 (19), 1350 (38), 1200 (53), 1350 (69), 1200 (83)**	**0.9546**	**413.67**
FHSA [44]	84, 85, 86, 87, 88, 89, 90, 91, 92, 93, 94, 95, 96	1350 (5), 150 (23), 1050 (29), 1350 (38), 900 (51), 1500 (71), 1200 (81)	0.9472	430.08
Case (iii): CB deployment after reconfiguration				
**GWO**	**55, 7, 86, 72, 13, 89, 90, 83, 92, 93, 34, 40, 62**	**1050 (6), 1200 (21), 1050 (28), 450 (38), 300 (50), 900 (60), 1350 (63), 1050 (71), 1050 (79)**	**0.9639**	**370.84**
Case (iv): simultaneous reconfiguration and CB deployment				
**GWO**	**84, 7, 86, 72, 76, 89, 90, 83, 92, 36, 34, 39, 62**	**1050 (7), 1050 (8), 1050 (17), 1350 (23), 1200 (37), 150 (50), 1200 (52), 1350 (71), 1350 (83)**	**0.9685**	**368.11**
FHSA [44]	7, 13, 33, 39, 42, 63,72, 84, 86, 89, 90, 91, 92	600 (6), 1350 (10), 1200 (21), 450 (36), 300 (42), 900 (53), 1500 (71), 450 (72), 1350 (82)	0.9640	375.71

**Table 9:** Scenario 3: Simultaneous reconfiguration with DG and Capacitor deployment for TPC 83-bus RDS.

Method	Switches opened	DG size, kW (bus number)	Capacitor size, kVAR (bus number)	Min. voltage (p.u.)	Power loss, kW
Base case	84, 85, 86, 87, 88, 89, 90, 91, 92, 93, 94, 95, 96	–	–	0.9285	531.99
Case (i): only reconfiguration					
**GWO**	**55, 7, 86, 72, 13, 89, 90, 83, 92, 93, 34, 40, 62**	**–**	**–**	**0.9531**	**470.10**
Case (ii): DG and CB deployment					
**GWO**	**84, 85, 86, 87, 88, 89, 90, 91, 92, 93, 94, 95, 96**	**2000 (6), 1816 (12), 2000 (21), 2000 (68), 1244 (83)**	**1500 (7), 450 (8), 900 (22), 750 (72), 1350 (83)**	**0.9600**	**290.35**
MG-TLBO [51]	84, 85, 86, 87, 88, 89, 90, 91, 92, 93, 94, 95, 96	2714 (10), 1892 (27), 2839 (51), 2134 (73), 1439 (74)	1000 (2), 2000 (8), 1500 (13), 1900 (78), 1300 (82)	0.9601	304.46
Case (iii): simultaneous reconfiguration with DG and CB deployment					
**GWO**	**84, 7, 86, 72, 88, 89, 90, 83, 92, 93, 34, 40, 63**	**1905 (8), 1083 (23), 2000 (55), 1702 (71), 1380 (80)**	**600 (6), 1500 (17), 1050 (19), 1500 (55), 1500 (69)**	**0.9653**	**252.97**

Table 9 presents the results for Scenario 3 (optimal reconfiguration with DG and CB deployment). For Case (ii) (DG and CB deployment), at 290.35 kW, the power loss for GWO is better than that of the other method which yields 304.46 kW. There are no results in the literature to compare with, for the last case of optimal reconfiguration with DG and CB deployment.

**Fig. 2:**
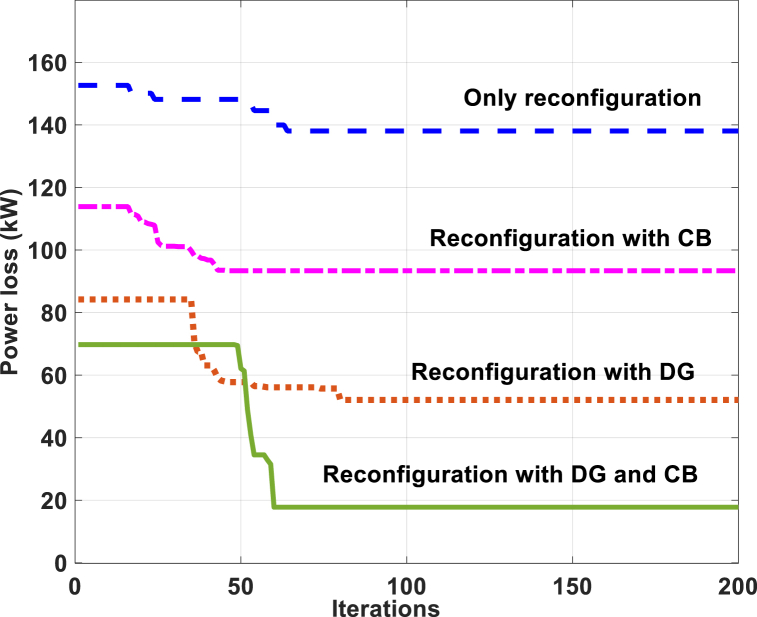
Power loss convergence characteristics for the IEEE 33-bus RDS by the GWO method.

**Fig. 3:**
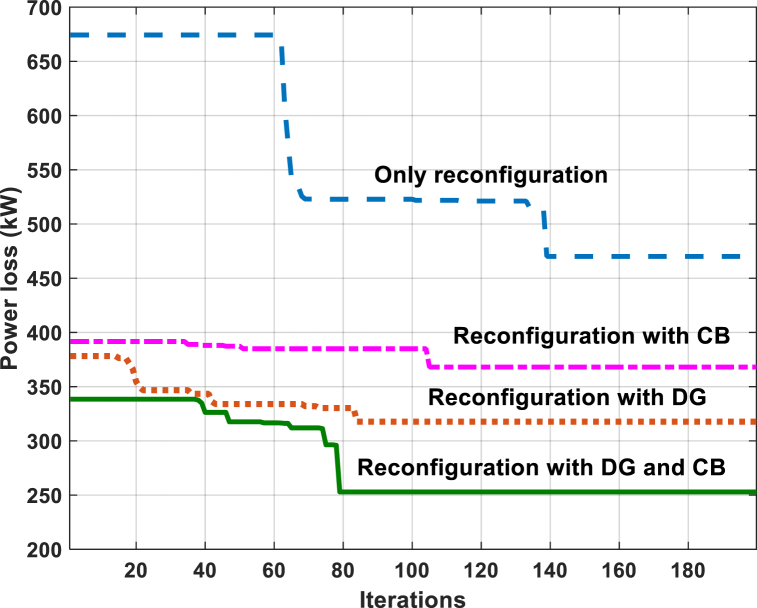
Power loss convergence characteristics for the IEEE 69-bus RDS by the GWO method.

**Fig. 4:**
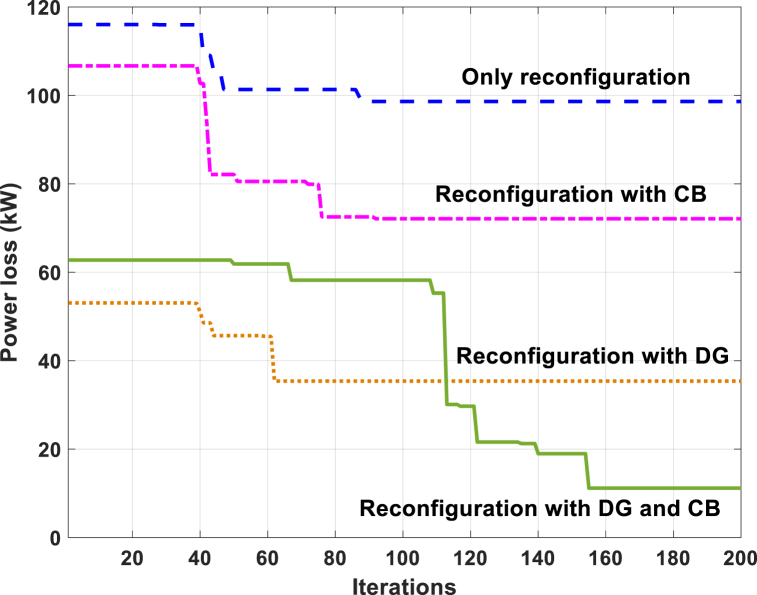
Power loss convergence characteristics for TPC 83-bus RDS by the GWO method.

An interesting comparison of the loss reduction through the iterations, of some of the Cases in the three Scenarios 1, 2 and 3 is presented in Fig. 2, Fig. 3, Fig. 4, for the IEEE 33- and 69- and TPC 83-bus RDSs. Expectedly, Scenario 3, Case (iii) (Simultaneous reconfiguration with DG and CB deployment) produces the lowest power loss for all the test cases.

**Fig. 5:**
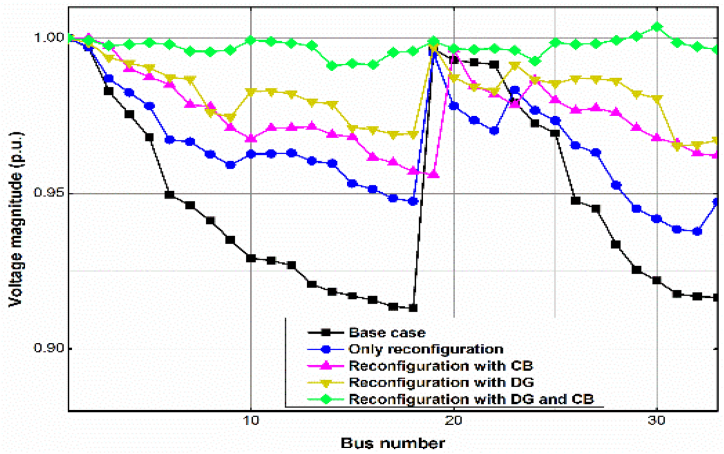
Voltage profile for the different Scenarios and Cases for the IEEE 33-bus RDS by the GWO method.

**Fig. 6:**
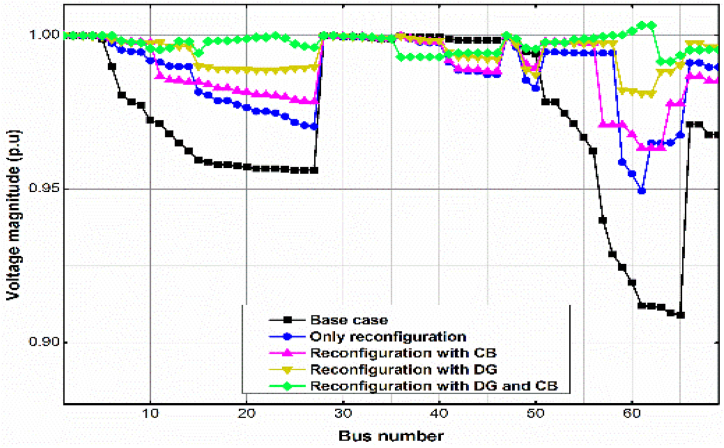
Voltage profile for the different Scenarios and Cases for the IEEE 69-bus RDS by the GWO method.

**Fig. 7:**
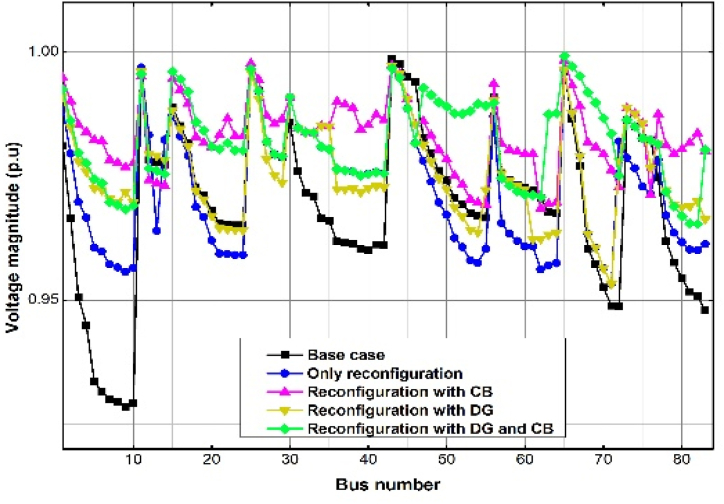
Voltage profile for the different Scenarios and Cases for TPC 83-bus RDS by the GWO method.

The voltage profile of the uncompensated case, and some of the Cases in the three Scenarios 1, 2 and 3 is presented in Fig. 5, Fig. 6, Fig. 7. As with the power loss, Scenario 3, Case (iii) produces the best, almost flat voltage profile at all the buses in all the test cases.

**Fig. 8:**
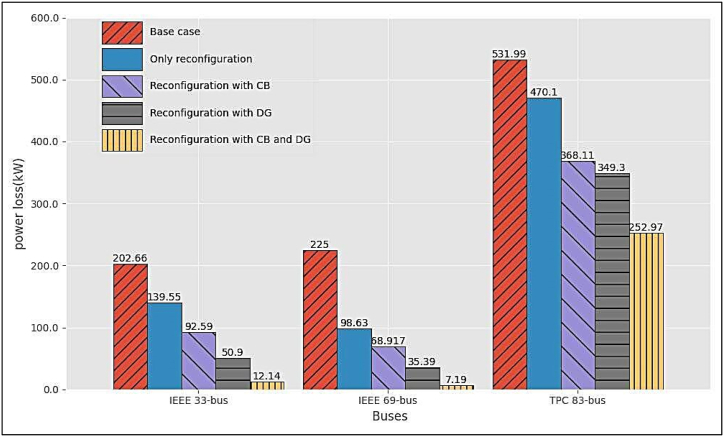
Comparison of power loss for the three RDSs by the GWO method.

Fig. 8 summarizes the comparison of the power losses for some of the Cases under Scenarios 1, 2 and 3, for all the test systems shown in the tables. Obviously, the best performance is obtained in Scenario 3, Case 3, for all the RDSs.

**Fig. 9:**
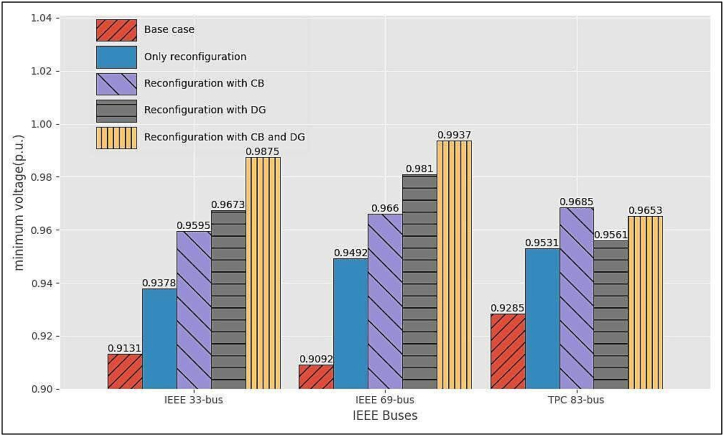
Comparison of minimum voltage for the three RDSs by the GWO method.

Fig. 9 summarizes the comparison of the improvement in the minimum voltages for some of the Cases under Scenarios 1, 2 and 3, for all the test systems shown in the tables and Fig.s 5, 6 and 7. Expectedly, the best performance is obtained in Scenario 3, Case 3, for all the RDSs.

## 6 Conclusion

A comparative study of the optimal reconfiguration, DG and CB deployment in a RDS has been presented in this paper. Numerous comparisons have been provided to establish the potency of the approach. Going further, the problem of simultaneous.

optimal reconfiguration, DG and CB deployment - a little studied problem in the literature - has been solved. The approach was demonstrated for IEEE 33-, 69- and TPC 83-bus systems.

The minimum power loss and improvement in the minimum voltage, arrived at by using the GWO is better than those by all the other methods in most of the scenarios and on par in a few scenarios for all the systems. The improvement in the overall performance proves that the GWO is a potent optimizer for dealing with optimization problems in distribution systems.

As compared to classical methods, metaheuristic methods - such as the GWO – “although do not guarantee optimality, are suitable to solve the RDS problem, especially for large-size systems” [67].

As the GWO requires almost no tuning - thus making it much simpler to implement compared with other metaheuristic and MINLP algorithms - it can be used for solving other challenging optimization problems.

Further scope for optimization involving energy storage may be explored [68,69]. Other metaheuristic methods too may be tried [[70], [71], [72], [73]].

## CRediT authorship contribution statement

**T. Jayabarathi:** Writing – review & editing, Writing – original draft, Validation, Supervision, Resources, Methodology, Investigation, Formal analysis, Data curation, Conceptualization. **T. Raghunathan:** Writing – review & editing, Writing – original draft, Validation, Software, Methodology, Investigation, Formal analysis. **N. Mithulananthan:** Visualization, Validation, Supervision, Methodology. **S.H.C. Cherukuri:** Software. **G. Loknath Sai:** Validation, Software.

## Declaration of competing interest

The authors declare that they have no known competing financial interests or personal relationships that could have appeared to influence the work reported in this paper.

## Abbreviations

3D-GSO: three-dimensional group search optimization
ACA: ant colony algorithm
ACSA: adaptive cuckoo search algorithm
BFO: bacterial foraging optimization
BPSO: binary search swarm optimization
COA: coyote algorithm
CSA: cuckoo search algorithm
CSGA: chaotic search group algorithm
EGA: enhanced genetic algorithm
FHSA: fuzzy harmony search algorithm
FWA: fireworks algorithm
GA: genetic algorithm
GA-ISEM: genetic algorithm-improved switch exchange method
GWO: grey wolf optimizer
HM: heuristic method
HSA: harmony search algorithm
ICA-GA: imperialist competitive algorithm and genetic algorithm
ICSA: improved cuckoo search algorithm
IEOA: improved equilibrium optimization algorithm
IMDE: intersect mutual differential evolution
INNA: improved neural network algorithm
ISCA: improved sine-cosine algorithm
ITSA: improved tunicate swarm algorithm
IWDA: intelligent water drop algorithm
MBA: theta-modified bat algorithm
MG-TLBO: modified group experience of teaching-learning-based optimization approach
MIHDE: mixed integer hybrid differential evolution
MINLP: mixed integer nonlinear programming
MRFOA: manta ray foraging optimization algorithm
NSGA-II: non-dominated sorting genetic algorithm – II
PGSA: plant growth simulation algorithm
PSO: particle swarm optimization
SFS: stochastic fractal search
UVDA: uniform voltage distribution based constructive reconfiguration algorithm
WCA: water cycle algorithm
